# The effectiveness of strategies to change organisational culture to improve healthcare performance: a systematic review

**DOI:** 10.1186/1748-5908-6-33

**Published:** 2011-04-03

**Authors:** Elena Parmelli, Gerd Flodgren, Fiona Beyer, Nick Baillie, Mary Ellen Schaafsma, Martin P Eccles

**Affiliations:** 1 Institute of Health and Society, Newcastle University, Baddiley-Clark Building, Richardson Road, Newcastle upon Tyne, NE2 4AX, UK; 2 Department of Oncology, Hematology and Respiratory Diseases, University of Modena and Reggio Emilia, Via del Pozzo 71, 41100 Modena, Italy; 3 National Institute for Health and Clinical Excellence, Level 1A, City Tower, Piccadilly Plaza, Manchester, M1 4BD, UK; 4 Canadian Cochrane Centre, 1 Stewart Street, Rm 227, Ottawa, ON K1N 6N5, Canada

## Abstract

**Background:**

Organisational culture is an anthropological metaphor used to inform research and consultancy and to explain organisational environments. In recent years, increasing emphasis has been placed on the need to change organisational culture in order to improve healthcare performance. However, the precise function of organisational culture in healthcare policy often remains underspecified and the desirability and feasibility of strategies to be adopted have been called into question. The objective of this review was to determine the effectiveness of strategies to change organisational culture in order to improve healthcare performance.

**Methods:**

We searched the following electronic databases: The Cochrane Central Register of Controlled Trials, MEDLINE, EMBASE, CINAHL, Sociological Abstracts, Web of Knowledge, PsycINFO, Business and Management, EThOS, Index to Theses, Intute, HMIC, SIGLE, and Scopus until October 2009. The Database of Abstracts of Reviews of Effectiveness (DARE) was searched for related reviews. We also searched the reference lists of all papers and relevant reviews identified, and we contacted experts in the field for advice on further potential studies. We considered randomised controlled trials (RCTs) or well designed quasi-experimental studies (controlled clinical trials (CCTs), controlled before and after studies (CBAs), and interrupted time series (ITS) analyses). Studies could be set in any type of healthcare organisation in which strategies to change organisational culture in order to improve healthcare performance were applied. Our main outcomes were objective measures of professional performance and patient outcome.

**Results:**

The search strategy yielded 4,239 records. After the full text assessment, two CBA studies were included in the review. They both assessed the impact of interventions aimed at changing organisational culture, but one evaluated the impact on work-related and personal outcomes while the other measured clinical outcomes. Both were at high risk of bias. Both reported positive results.

**Conclusions:**

Current available evidence does not identify any effective, generalisable strategies to change organisational culture. Healthcare organisations considering implementing interventions aimed at changing culture should seriously consider conducting an evaluation (using a robust design, *e.g.*, ITS) to strengthen the evidence about this topic.

## Background

Organisational culture is an anthropological metaphor used to inform research and consultancy and to explain organisational environments [1]. Several definitions of organisational culture can be found in literature [2]. They range from the extremely simple -- 'the way we do things around here' [3] -- to the more complex such as that proposed by Schien: 'the pattern of shared basic assumption -- invented, discovered or developed by a given group as it learns to cope with its problems of external adaptation and internal integration -- that has worked well enough to be considered valid and therefore to be taught to new members as the correct way to perceive, think and feel in relationship to those problems' [4]. What appears to be consistent through all these definitions is that the term organisational culture pertains to the multiple aspects of what is shared among people within the same organisation: for example beliefs, values, norms of behaviour, routines, traditions, sense-making, *et al*. Culture is therefore a lens through which an organisation can be understood and interpreted [5]. Scott *et al. *in 2003 [6] highlighted that culture is not merely the observable in social life, but also the shared cognitive and symbolic context within which a society can be understood. For this reason, they decided to adopt Schien's definition that seemed to better include all the different aspects of organisational culture. For this review we have chosen to do the same.

Increasing emphasis has been placed during recent years on the need to change organisational culture alongside structural reforms in order to pursue effective improvement of healthcare performance [7-9]. However, the management of culture change is a complicated task; its precise function in healthcare policy often remains underspecified and the desirability and feasibility of strategies to be adopted have been called into question [10].

A survey conducted in 275 English National Health Service (NHS) organisations in 2008 [1] highlighted that one-third of them currently used a culture assessment instrument to support their clinical governance activity, although most of this use related to one instrument (Manchester Patient Safety Framework [11]). Within this survey [1], Mannion *et al. *reviewed the literature about instruments available to health services researchers wishing to measure culture and culture change. They identified two-dozen tools used for culture assessment and having potential relevance to healthcare organisations; relatively few of these had been used to any extent in the NHS. Extant tools covered many of the most important organisational culture attributes, but their focus in use was on safety rather than on the assessment of dimensions of healthcare quality and performance. Moreover, little evaluation of the use and the practical application of these tools or how well they connect with ongoing policy, managerial, or service preoccupations is available. A similar message came from a more recent review in which Jung *et al. *[12] identified 70 qualitative or quantitative instruments for exploring organisational culture for formative, summative, or diagnostic reasons. They described the majority as 'at a preliminary stage of development' and concluded that there was 'no ideal instrument for cultural exploration.'

The idea that organisational culture can affect performance is based in particular on the assumption that they are related, but evidence from the research literature for this link is weak [13]. A review conducted by Scott *et al. *focused on this relationship. They qualitatively summarised ten empirical studies investigating the relationship between culture and performance and concluded that 'there is some evidence to suggest that organisational culture may be a relevant factor in healthcare performance, yet articulating the nature of that relationship proves difficult' [6]. More recently, Mannion *et al. *compared, in a multiple case study design, the cultural characteristics of 'high' and 'low' performing hospitals in the UK NHS [14]. They found that different cultural patterns could be identified within cases grouped by performance, and concluded that organisational culture is associated with performance, but they highlighted that the interpretation of their results should be tempered with a degree of caution because of some methodological issues.

Nonetheless, the management of organisational culture is increasingly viewed as a necessary part of health system reform [15-17]. In 2008, a survey conducted across a total of 325 English NHS primary and acute trusts reported that 98% of responding clinical governance managers saw the need to measure local culture in order to foster change for improved performance; nearly all of them (99%) acknowledged the importance of understanding and shaping local cultures, but the majority (88%) were also conscious that there are many challenges to overcome to implement and sustain beneficial culture change [5]. It is therefore timely and important to review the literature on the effectiveness of strategies to change organisational culture in order to improve healthcare performance.

The objectives of this review were: to determine the effectiveness of strategies to change organisational culture in improving healthcare performance and to examine the effectiveness of these strategies according to different patterns of organisational culture.

## Methods

We considered randomised controlled trials (RCTs) or well designed quasi-experimental studies, controlled clinical trials (CCTs), controlled before and after studies (CBAs), and interrupted time series (ITS) analyses set in any type of healthcare organisation and investigating strategies to change organisational culture in order to improve healthcare performance. ITS analyses were eligible if they had a clearly defined point in time when the intervention occurred and three data collection points before and after the intervention to take into account secular trends and auto-correlation among measurements over time [18].

The two main outcomes of the review were: objective measures of professional performance such as prescription rates, the extent to which care is evidence based, quality of care; and objective measures of patient outcome such as mortality (standardised mortality ratio), condition-specific measures of outcome, quality of life, functional health status, and patients' satisfaction.

We also report other included outcomes such as: objective measures of organisational performance (such as wait times, inpatient hospital stay times, and staff turnover rates); measures of organisational culture; economic outcomes (such as efficiencies and changes in costs); and measures of health practitioners' knowledge, attitudes, satisfaction.

To identify studies eligible for this review we searched the following electronic databases for primary studies: The Cochrane Central Register of Controlled Trials (*The Cochrane Library *2009, Issue 4), MEDLINE - Ovid (1950 to October Week 3 2009), EMBASE - Ovid (1980 to 2009 Week 41), CINAHL - EBSCO (1980 to October 2009), Sociological Abstracts - CSA (1952 to October 2009), Social Science Citation Index - Web of Knowledge (1970 to October 2009), Science Citation Index - Web of Knowledge (1970 to October 2009), Conference Proceedings - Web of Knowledge (1970 to October 2009), PsycINFO - Ovid (1806 to October Week 3 2009), Business and Management - OCLC FirstSearch (1995 to October 2009), EThOS (British Library), Index to Theses (1716 to October 2009), Intute, HMIC - Ovid (1979 to October 2009), SIGLE, Scopus (1823 to October 2009). Search strategies for primary studies incorporated the methodological component of the Cochrane Collaboration Effective Practice and Organisation of Care Review Group search strategy combined with selected index terms and free text terms. We translated the MEDLINE search strategy into the other databases using the appropriate controlled vocabulary as applicable. The full search strategies are presented in Additional File 1. We also searched the reference lists of all papers and relevant reviews identified, and we contacted experts in the field for advice on further potential studies. Finally, we searched the Database of Abstracts of Reviews of Effectiveness (DARE) for related reviews.

We downloaded all titles and abstracts retrieved by electronic searching to the reference management database EndNote, and removed duplicates. At least two review authors (from EP, GF, MPE) independently examined the remaining references. We excluded those studies that clearly do not meet the inclusion criteria and obtained copies of the full text of potentially relevant references. At least two review authors (from EP, GF, MES, MPE, NB) independently assessed the eligibility of retrieved papers and extracted the data using a specifically developed checklist. We used the same criteria as those outlined in the *Cochrane Handbook for Systematic Reviews of Interventions *to evaluate data [19] and we resolved any disagreement by discussion and the involvement of an arbitrator (MPE) as necessary.

The risk of bias of the eligible studies was evaluated independently by at least two reviewers using the following criteria: RCTs, CCTs, and CBAs were assessed for generation of allocation sequence, concealment of allocation, baseline outcome measurements, baseline characteristics, incomplete outcome data, blinding of outcome assessor, protection against contamination, selective outcome reporting, and other risks of bias. ITS designs were also assessed for the independence of the intervention from other changes, the pre-specified shape of the intervention, and whether the intervention was likely or unlikely to affect data collection. Data were reported in natural units. Where baseline results were available from RCTs, CCTs, and CBAs, we reported pre-intervention and post-intervention means or proportions for both study and control groups. We calculated the adjusted (for any baseline imbalance) absolute change from baseline reported as the adjusted risk difference (ARD) calculated as: (Intervention Follow-up - Intervention Baseline) - (Control Follow-up - Control Baseline).

## Results

**Figure 1:**
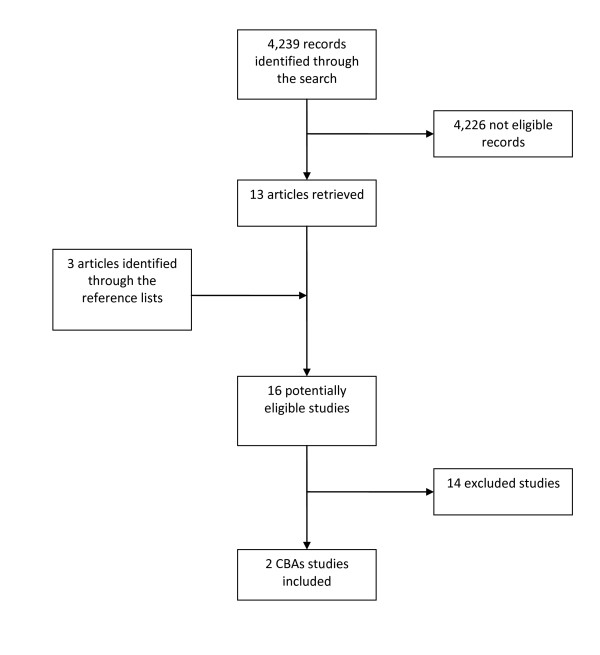
**Flowchart of the review**. Flowchart of the searched and retrieved reference for the review.

The search strategy identified 4,239 records. After the independent examination by the reviewers, we retrieved 13 articles potentially eligible for the review. Three more articles were identified from the reference lists of those retrieved. After full text assessment, two studies [20,21] met the inclusion criteria (Figure 1). For a description of excluded studies and reasons for their exclusion see Additional File 2; of 14 studies, six were not aiming to change organisational culture, two reported self-report outcome measures only (and were not measuring organisational culture), and six used designs that were excluded by the review criteria. The characteristic of the two included studies are reported in Table 1. Both of them used a CBA design to assess the impact of interventions aimed at changing organisational culture; Kinjerski [20] evaluated the impact on work-related and personal outcomes while Larson [21] measured clinical outcomes; both were at high risk of bias (see Table 1). They both report positive results (see Tables 2 and 3).

**Table 1:** Characteristics of included studies

	Larson	Kinjerski
**Study design**	CBA	CBA

**Providers**	Manager, medical and nurse leaders	RNs; LPNs; RNAs; other (admin, housekeeping, food service, physio)

**Patients**	Adult and neonatal	Elderly long-term care residents

**Setting**	Two hospitals in mid-Atlantic region	Two long-term care units, Canada
**Unit of allocation**	Adult intensive care unit (ICU) and neonatal ICU	Long-term care unit

**Unit of analysis**	Hospital	Long-term care unit

**Intervention**	Top-level administrative intervention using a framework for changing organisational culture. Interventions included dissemination of key messages, marketing approaches (distribution of samples), education interventions, audit and feedback, opinion leaders (supervisors).	Organizational intervention through education sessions to 'boost morale' and improve provider satisfaction with their work, offering psychic rewards. - 1-day workshop on 'cultivating spirit at work in long-term care' - 1-hour booster sessions each week at shift changes

**Control**	Standard care	Standard care

**Target behaviour**	Handwashing practice	Employee spirit at work, employee wellness, job satisfaction, organizational commitment, turnover, absenteeism.

**Outcomes**	a) Handwashing frequency b) Nosocomial infection associated with methicillin-resistant Staphylococcus aureus (MRSA) and vancomycin-resistant enterococci (VRE).	a) Health professional outcomes/process measures: decrease in turnover and absenteeism; improved employee spirit at work, employee wellness, job satisfaction and organizational commitment. b) Patient outcomes: increased focus on residents with implications for Quality of Care (not stated as an outcome to be measured, but reported on as a result of the program).

**Risk of Bias assessment**		

Allocation sequence adequately generated	NO	NO

Allocation adequately concealed	NO	NO

Baseline outcome measurements similar	NO	YES

Baseline characteristics similar	UNCLEAR	UNCLEAR

Incomplete outcome data adequately addressed	YES	YES

Knowledge of the allocated interventions adequately prevented	NO	NO

Protection against contamination	UNCLEAR	UNCLEAR

Free from selective outcome reporting	YES	UNCLEAR

Free from other risks of bias	NO (one site CBA)	NO (one site CBA)

**Table 2:** Results for Larson 2000

Outcomes		Comparison		Intervention		ARD	RR (95% CI)		Ratio of change (baseline - follow-up)	
				
		Baseline	Follow-up	Baseline	Follow-up		Baseline	Follow-up	Comparison	Intervention
**Frequency of handwashing**	N° soap-dispensing episodes/patient-care days	30.3	55.5	42.6	116.6	48.8	1.4 (1.3 to 1.52)	2.1 (1.99 to 2.21)	-	-

MRSA*	Incident density/1,000 patient-care days	0.385	0.503	0.464	0.309	0.273	1.21 (0.63 to 2.32)	0.61 (0.31 to 1.21)	0.181 (31% increase)	0.07 (33% decrease)

VRE**	Incident density/1,000 patient-care days	0.700	0.394	0.464	0.070	0.088	0.66 (0.38 to 1.14)	0.19 (0.04 to 0.65)	0.56 (44% decrease)	0.15 (85% decrease)

* methicillin-resistant *Staphylococcus aureus*

** vancomycin-resistant *enterococci*

**Table 3:** Results (Means and ANOVA) for Kinjerski 2008

		**Comparison**^1^		**Intervention**^1^		ARD	Main Effect		Interaction
				
Outcomes	Instruments	Pretest	Posttest	Pretest	Posttest		Group	Time	Group by Time
**Work-related outcomes**									

Spirit at work	The Spirit at Work Scale 18 items (1 → 6)	85.6	84.5	81.2	90.5	10.4	F < 1	F(1.49) = 8.62**	F(1.49) = 13.88***
Job satisfaction	The Job Satisfaction Scale 14 items (1 → 7)	81	77.8	69.7	76.4	9.9	F(1.40) = 4.94*	F < 1	F(1.40) = 7.25**
Organisational commitment	The Organisational Commitment Scale 15 items (1 → 7)	49.3	48.3	45.2	51.1	6.9	F < 1	F(1.50) = 4.20*	F(1.50) = 8.27**
Organisational culture	The Organisational Culture Survey 31 items, 6 areas (1 → 5)	116.8	116.7	101.7	115.3	13.7	F(1.42) = 4.24*	F(1.42) = 7.20*	F(1.42) = 7.56**
Team work	The Organisational Culture Survey (1 → 5)	20.8	20.8	17.5	21.5	4	F(1.49) = 2.22	F(1.49) = 9.76**	F(1.49) = 10.49**
Morale/climate	The Organisational Culture Survey (1 → 5)	18.8	19.2	16.8	19.7	3.6	F < 1	F(1.49) = 10.52**	F(1.49) = 5.88*
**Personal outcomes**									
Vitality	The Vitality Scale 7 items (1 → 7)	37	37	35.8	37.3	1.5	F < 1	F(1.50) = 1.06	F < 1
Life satisfaction	Satisfaction with Life Scale 5 items (1 → 7)	26.5	28.1	27	29.8	1.2	F < 1	F(1.49) = 10.25**	F < 1
Orientation to life	Sense of Coherence Scale 13 items (1 → 7)	67.3	68.8	62.8	66.8	2.5	F(1.48) = 1.56	F(1.48) = 4.28*	F < 1

^1 ^Mean scores: higher score = better outcomes

*p < 0.05; *p < 0.01; ***p < 0.001

Larson *et al. *[21] introduced a top-level administrative intervention using a framework for changing organisational culture on staff handwashing frequency; the purpose of the study was to measure the impact of the intervention on handwashing frequency and rates of selected nosocomial infections. The study took place in two hospitals (one serving as an intervention site and the other as control) in the mid-Atlantic region of the USA; they had similar infection prevention and control programmes. A two-tiered strategy for the administrative intervention was developed and implemented based on Schien's framework for changing organisational culture [4] that suggested that leaders have the greatest potential for reinforcing new aspects of culture. First, top management and medical and nursing leaders agreed to provide active support for a culture change that would highlight and enforce the expectations for handwashing compliance for all healthcare workers. Second, managers responsible for implementation were given an opportunity to develop the specific elements of the intervention. This resulted in a composite intervention consisting of educational programs, information materials, distribution of handwashing fact sheets and hand-hygiene products samples, and supervisory/supporting activities. Rates of nosocomial infection were calculated for both of the study hospitals as the number of cases per 1,000 patient-care days. Surveillance methods were the same in both hospitals. A surrogate for handwashing frequency was measured using counting devices placed inside every soap dispenser of four selected units (two in each hospital). In the intervention hospital, the mean handwashing frequency per patient-care day measured after six months of follow-up was higher than in the control hospital (see Table 2), but it is unclear if the analysis has taken account of the baseline imbalance. No statistically significant difference was found in methicillin-resistant *Staph. aureus *(MRSA) rates between the two hospitals during the follow-up phase, but the intervention hospital showed significantly lower rates of vancomycin-resistant *enterococci *(VRE) (RR = 0.19, p = 0.002).

Kinjerski and Skrypnek [20] explored whether a 'spirit at work' intervention program could increase employee spirit at work, employee wellness, job satisfaction, and organizational commitment, and decrease absenteeism and turnover. The intervention consisted of a one day workshop, 'Cultivating Spirit at Work in Long-Term Care,' supplemented by eight weekly one hour booster sessions. The workshop focused on spirit at work -- what it is, personal strategies to foster it (*i.e.*, living purposely, living spiritually, appreciating self and others, and refilling the cup), and organizational conditions to cultivate it (*e.g.*, inspired leadership, sense of community, personal fulfilment, positive workplace culture). Participants were led through a variety of exercises that culminated in the creation of personal action plans to enhance spirit at work. Booster sessions were offered each week before and after shift change. The results show significant changes in six of the nine worker completed measures, including a measure of organisational culture (Table 3). Absenteeism rates (the per cent sick/paid hours in five months after the workshop compared with the same five months in the previous year) were no different pre-intervention (4.2% intervention group, 4.1% control group, Chi^2 ^<1, ns). The post-intervention difference was significant (1.7% intervention group, 3.5% control group, Chi^2 ^= 127.82, *df *= 1, p < 0.001). Turnover rates (per cent unit staff leaving/total staff on the unit over eight months pre- and five months post-introduction of the program) were no different pre-intervention (10.5% intervention group, 9.8% control group, Chi^2 ^<1, ns). The post-intervention difference was significant (2.6% intervention group, 16.4% control group, Chi^2 ^= 4.49, *df *= 1, p < 0.05). None of the analyses were reported as adjusting for baseline imbalance.

## Discussion

We identified two studies that evaluated the effects of interventions aimed at changing organisational culture. Both studies reported positive effects -- one on behavioural and clinical measures, and the other on study subject reported outcome measures and two indicators of organisational performance. Whilst this may seem encouraging, there are a number of methodological issues suggesting that these results should be treated with caution.

Both studies used a controlled before-after design, with one site experiencing the intervention and one site acting as control. Therefore any intervention effect is confounded by a possible (unknown) site effect. If researchers are evaluating interventions to change organisational culture and wish to produce generalisable findings, there is no reason why they should not use designs that would allow general inferences to be made with more confidence than is possible with the currently reported studies. In addition, neither study seemed to have allowed for the apparent baseline imbalance between their groups when calculating their effect sizes.

Both studies delivered complex interventions. One study [21] set out to change organisational culture and used an appropriate framework to do so but did not report any measure of organisational culture within the study. This means that it is not possible to understand if the intervention managed to change the organisational culture. In addition, this study delivered their 'culture changing' intervention to senior and middle managers, the latter of whom then developed and delivered a series of different interventions (many of which have evidence of their ability to change behaviour in their own right), and so it is not possible to disentangle the active ingredients within what was delivered. The second study [20] set out to change spirit at work but did measure, and reported a change in, organisational culture within this context. It is not clear how much of the intervention was specifically aimed at changing organisational culture (and so could be considered for examination in other studies) and how much of the effect was just a by-product of an intervention aimed primarily at a different concept. Finally, neither study provided a comprehensive description of activities in the control group as is recommended [22] in order to facilitate interpretation of intervention effects.

It is important to consider possible reasons for why this review included only two controlled studies of culture change interventions. Whilst using well-recognised systematic review methods, the construction of the search strategy was difficult; we included terms related to culture (and also allowed the term 'climate' though we excluded the term 'safety') resulting in a broad search that had to be manually sifted by two of authors. It is possible that we missed studies within this process. The review would also miss unpublished studies and so publication bias remains a threat to the findings of the review.

Studies of organisational culture are most commonly found in the organisational and management research literature rather than the biomedical literature. Organisational research has context and methodological norms that differ from those of biomedicine and so trials are rare and the epistemological and methodological assumptions are different from the norms of science -- as exemplified in a review by Jung *et al. *of organisational culture measurement instruments [12]. So, whilst there are those who seek to diagnose and subsequently change organisational culture to align it with that of highly performing organisations, they are unlikely to conduct such work within the designs included within this review. We have conducted this review using our criteria of methodological validity and are aware that these may be contested by some readers of this review. Although our perspective will have driven our sifting of the literature search, we still only identified 16 studies and only excluded six of these on our design criteria. Even had we considered these and they had all been positive, eight studies would still reflect a small and uncertain body of evidence. Given the limitations in the available evidence, and in the light of the considerable health service interest in the use of measures for organisational culture, research efforts should focus on generating evidence about the effectiveness of methods to change organisational culture to improve healthcare performance. However, given the multiplicity of measures [1,12], it may be the case that researchers need to continue to work to establish a clear definition of organisational culture and agree on reliable methods of measuring it.

At the moment the available evidence does not identify any effective, generalisable strategies to change organisational culture, and healthcare organisations considering implementing interventions aimed at changing culture should seriously consider conducting an evaluation (using a robust design, *e.g.*, ITS) to strengthen the evidence about this topic.

## Conclusions

No conclusions can be made about the effectiveness of strategies to change organisational culture to improve healthcare performance as high quality evidence on the effectiveness of strategies to change organisational culture is lacking. Researchers wishing to evaluate the effectiveness of strategies to change organisational culture should conduct evaluations using appropriately robust designs if the intent is to offer generalisable findings.

## Conflict of interests

MPE is Co-Editor in Chief of Implementation Science. All decisions on this manuscript were made by another editor.

## Authors' contributions

MPE conceived of the idea for the review. EP wrote the protocol and led the writing of the manuscript. All authors contributed to the literature sifting, data extraction, and writing. All authors approved the final submitted version of the manuscript.

## Supplementary Material

Additional File 1 **Search Strategies**. Full search strategies Click here for file

Additional File 2 **Excluded studies**. Excluded studies with reasons for exclusion Click here for file
