# Supplementary material for: The effectiveness of strategies to change organisational culture to improve healthcare performance: a systematic review
Source: Implement Sci. 2011 Apr 3;6:33. doi: 10.1186/1748-5908-6-33 (PMC3080823; doi:10.1186/1748-5908-6-33)
Supplement: Additional File 1 — Search Strategies. Full search strategies [file 1748-5908-6-33-S1.DOC]

**Additional File 1. Search Strategy**

**Cochrane Library**

1. Organizational culture/

2. (organisation* NEAR/5 cultur*):ti or (organization* NEAR/5 cultur*):ti or (organisation* NEAR/5 cultur*):ab or (organization* NEAR/5 cultur*):ab

3. (corporate NEXT culture* or workplace NEXT culture* or work NEXT culture* or organsation* NEXT ethos or organization* NEXT ethos or organisation* NEXT climate* or organization* NEXT climate*):ti or (corporate NEXT culture* or workplace NEXT culture* or work NEXT culture* or organsation* NEXT ethos or organization* NEXT ethos or organisation* NEXT climate* or organization* NEXT climate*):ab

4. 1 or 2 or 3

**MEDLINE**

1. Organizational culture/

2. (organi?ation$ adj5 cultur$).ti,ab.

3. (corporate culture? or workplace culture? or work culture? or organ?ation$ ethos or organi?ation$ climate?).ti,ab

4. 1 or 2 or 3

5. randomized controlled trial.pt.

6. random$.tw.

7. intervention$.tw.

8. control$.tw.

9. evaluat$.tw.

10. or/1-5

11. Animals/

12. Humans/

13.11 not (11 and 12)

14. 10 not 13

15. 4 and 14

**EMBASE**

1. (organi?ation$ adj5 cultur$).ti,ab.

2. (corporate culture? or workplace culture? or work culture? or organ?ation$ ethos or organi?ation$ climate?).ti,ab

3. 1 or 2

4. randomized controlled trial/

5. (randomised or randomized).tw.

6. experiment$.tw.

7. (time adj series).tw.

8. (pre test or pretest or post test or posttest).tw.

9. impact.tw.

10. intervention?.tw.

11. chang$.tw.

12. evaluat$.tw.

13. effect?.tw.

14. compar$.tw.

15. or/1-11

16. nonhuman/

17. 15 not 16

18. 3 and 17

**CINAHL**

Organizational culture/ NT Organizational Politics

1. (MH "Organizational Culture+")

2. TI (organi?ation* N5 cultur*) or AB (organi?ation* N5 cultur*)

3. TI (corporate culture* or workplace culture* or work culture* or organ?ation* ethos or organi?ation* climate*) or AB (corporate culture* or workplace culture* or work culture* or organ?ation* ethos or organi?ation* climate*)

4. 1 or 2 or 3

5. (MH "Clinical Trials+") or (MH "Comparative Studies") or (MH "Pretest-Posttest Design") or (MH "Quasi-Experimental Studies+")

6. TI (control* or random* or experiment or time series or impact or intervention? or evaluat* or effect?) or AB (control* or random* or experiment or time series or impact or intervention? or evaluat* or effect?)

7. 5 or 6

8. 4 and 7

**Sociological Abstracts**

1. DE=(“Organizational culture”)

2. TI=(organi?ation* WITHIN 5 cultur*) or AB=(organi?ation* WITHIN 5 cultur*)

3. TI=(corporate culture* or workplace culture* or work culture* or org

an?ation* ethos or organi?ation* climate*) or AB=(corporate culture* or workplace culture* or work culture* or organ?ation* ethos or organi?ation* climate*)

4. 1 or 2 or 3

5. DE=("nurses" or "health professions" or "physicians" or "dentists" or exp "health" or exp "health care services" or "pharmacists" or "psychiatrists" or "psychologists")

6. TI=(health or hospital* or “primary care” or “primary health care” or nurse* or doctor* or GP or physician* or clinician* or dentist* or dental or gyn?ecologist* or h?ematologist* or internist* or obstetrician* or p?ediatrician* or pharmacist* or physiotherapist* or psychiatrist* or psychologist* or radiologist* or surgeon* or surgery or therapist* or counse?lor* or neurologist* or optometrist*) or AB=(health or hospital* or “primary care” or “primary health care” or nurse* or doctor* or GP or physician* or clinician* or dentist* or dental or gyn?ecologist* or h?ematologist* or internist* or obstetrician* or p?ediatrician* or pharmacist* or physiotherapist* or psychiatrist* or psychologist* or radiologist* or surgeon* or surgery or therapist* or counse?lor* or neurologist* or optometrist*) (terms taken from REBEQI search)

7. 5 or 6

8. TI=(randomi?ed or experiment* or impact* or intervention* or evaluat* or effect* or comparative or pre test or pretest or posttest or post test) or AB=(randomi?ed or experiment* or impact* or intervention* or evaluat* or effect* or comparative or pre test or pretest or posttest or post test)

9. TI=(time WITHIN 2 series) or TI=(random* WITHIN 2 allocat*) or TI=(random* WITHIN 2 assign*) or TI=(controlled WITHIN 2 trial*) or TI=(controlled WITHIN 2 study) or AB=(time WITHIN 2 series) or AB=(random* WITHIN 2 allocat*) or AB=(random* WITHIN 2 assign*) or AB=(controlled WITHIN 2 trial*) or AB=(controlled WITHIN 2 study)

10. 8 or 9

11. 4 and 7 and 10

**Web of Knowledge: SCI, SSCI, Conference Proceedings**

1. TS=(organisation* SAME cultur*) or TS=(organization* SAME cultur*)

2. TS=("corporate culture*" or "workplace culture*" or "work culture*" or "organization* ethos" or "organisation* ethos" or "organization* climate*" or "organisation* climate*")

3. 1 or 2

4. TS=(health OR hospital* OR nurse* OR doctor* OR GP OR physician* OR clinician* OR dentist* OR dental OR gyn$ecologist* OR h$ematologist* OR internist* OR obstetrician* OR p$ediatrician* OR pharmacist* OR physiotherapist* OR psychiatrist* OR psychologist* OR radiologist* OR surgeon* OR surgery OR therapist* OR counsel$or* OR neurologist* OR optometrist OR “primary care” OR “primary health care”)

5. TS=(randomi?ed or experiment* or impact* or intervention* or evaluat* or effect* or comparative or "time series")

6. TS=(random* SAME allocat*) or TS=(random* SAME assign*) or TS=(controlled SAME trial*) or TS=(controlled SAME study)

7. 5 or 6

8. 3 and 4 and 7

**PsycINFO**

1. Organizational climate/

2. (organi?ation$ adj5 cultur$).ti,ab.

3. (corporate culture? or workplace culture? or work culture? or organ?ation$ ethos or organi?ation$ climate?).ti,ab

4. 1 or 2 or 3

5. (randomi?ed or experiment* or impact* or intervention* or evaluat* or effect* or comparative or pre test or pretest or posttest or post test).tw

6. ((time adj2 series) or (random* adj2 allocat*) or (random* adj2 assign*) or (controlled adj2 trial*) or (controlled adj2 study)).tw

7. 5 or 6

8. exp Health Care Services/

9. (health care or primary care or hospital* or surgery or surgeries).tw

10. exp Health Personnel/ or exp Clinicians/ or exp Counselors/ or exp Therapists/ or exp Social Workers/

11. (nurse* or doctor* or GP or physician* or clinician* or dentist* or dental or gyn?ecologist* or h?ematologist* or internist* or obstetrician* or p?ediatrician* or pharmacist* or physiotherapist* or psychiatrist* or psychologist* or radiologist* or surgeon* or surgery or therapist* or counsel?or* or neurologist* or optometrist*).tw

12. Or/8-11

13. 4 and 7 and 12

**BusManagement**

1. kw: organi#ation* n5 culture+ or kw: corporate w culture+ or kw: workplace w culture+ or kw: work w culture+ or kw: organi#ation* w ethos or kw: organi#ation* w climate+

2. kw: health w2 trust OR kw: hospital+ OR kw: nurse+ OR kw: doctor+ OR kw: GP OR kw: physician+ OR kw: clinician+ OR kw: dentist+ OR kw: dental OR kw: gynecologist+ OR kw: gynaecologist+ OR kw: hematologist+ OR kw: haematologist+ OR kw: internist+ OR kw: obstetrician+ OR kw: pediatrician+ OR kw: paediatrician+ OR kw: pharmacist+ OR kw: physiotherapist+ OR kw: psychiatrist+ OR kw: psychologist+ OR kw: radiologist+ OR kw: surgeon+ OR kw: surgery OR kw: therapist+ OR kw: counselor+ OR kw: counsellor+ OR kw: neurologist+ OR kw: optometrist OR kw: primary w2 care

3. kw: randomly or kw: randomi* or kw: factorial* or kw: controlled w2 trial* or kw: evaluat* or kw: trial* or kw: experiment* or kw: study or kw: studies or kw: design or kw: crossover* or kw: cross-over* or kw: cross w over or kw: placebo* or kw: assign* or kw: volunteer* or kw: intervention* or kw: effect* or kw: compar* or kw: impact+ or kw: time w series

4. 1 and 2 and 3

**EThOS (theses)**

"Organisational culture" or "organizational culture"

**Index to Theses**

(organi?ation w/5 cultur*) and health

**Intute**

Organisational culture or organizational culture in any field.

**HMIC**

organisational culture and methods (as psycinfo)

**SIGLE**

(("organisational culture" OR "organizational culture") AND (health OR care))

**Scopus**

#1 INDEXTERMS(**organi?ational** **culture**))

#2 TITLE((**organisation*** W/5 **culture***) OR (**organization*** W/5 **culture***) OR (**corporate** **culture***) OR (**workplace** **culture***) OR (**work** **culture***) OR (**organisation*** **ethos**) OR (**organization*** **ethos**) OR (**organisation*** **climate***) OR (**organization*** **climate***))

#3 ABS((**organisation*** W/5 **culture***) OR (**organization*** W/5 **culture***) OR (**corporate** **culture***) OR (**workplace** **culture***) OR (**work** **culture***) OR (**organisation*** **ethos**) OR (**organization*** **ethos**) OR (**organisation*** **climate***) OR (**organization*** **climate***))

#4 #1 OR #2 OR #3

#5 TITLE(**health** OR **hospital*** OR **nurse*** OR **doctor*** OR **gp** OR **physician*** OR **clinician*** OR **dentist*** OR **dental** OR **gynecologist*** OR **gynaecologist*** OR **hematologist*** OR **haematologist*** OR **internist*** OR **obstetrician*** OR **pediatrician*** OR **paediatrician*** OR **pharmacist*** OR **physiotherapist*** OR **psychiatrist*** OR **psychologist*** OR **radiologist*** OR **surgeon*** OR **surgery** OR **therapist*** OR **counselor*** OR **counsellor*** OR **neurologist*** OR **optometrist** OR **"primary care"** OR **"primary health care"**)

#6 ABS(**health** OR **hospital*** OR **nurse*** OR **doctor*** OR **gp** OR **physician*** OR **clinician*** OR **dentist*** OR **dental** OR **gynecologist*** OR **gynaecologist*** OR **hematologist*** OR **haematologist*** OR **internist*** OR **obstetrician*** OR **pediatrician*** OR **paediatrician*** OR **pharmacist*** OR **physiotherapist*** OR **psychiatrist*** OR **psychologist*** OR **radiologist*** OR **surgeon*** OR **surgery** OR **therapist*** OR **counselor*** OR **counsellor*** OR **neurologist*** OR **optometrist** OR **"primary care"** OR **"primary health care"**)

#7 #5 OR #6

#8 TITLE(**randomly** OR **randomi*** OR **factorial*** OR **"controlled trial"** OR **evaluat*** OR **trial*** OR **experiment*** OR **study** OR **studies** OR **design** OR **crossover*** OR **cross-over*** OR **“cross** **over”** OR **placebo*** OR **assign*** OR **volunteer*** OR **intervention*** OR **effect*** OR **compar*** OR **impact** OR **“time** **series”**)

#9 ABS(**randomly** OR **randomi*** OR **factorial*** OR **"controlled trial"** OR **evaluat*** OR **trial*** OR **experiment*** OR **study** OR **studies** OR **design** OR **crossover*** OR **cross-over*** OR **“cross** **over”** OR **placebo*** OR **assign*** OR **volunteer*** OR **intervention*** OR **effect*** OR **compar*** OR **impact** OR **“time** **series”**)

#10 #8 OR #9

#11 #4 AND #7 AND #10
