# Supplementary material for: The effectiveness of strategies to change organisational culture to improve healthcare performance: a systematic review
Source: Implement Sci. 2011 Apr 3;6:33. doi: 10.1186/1748-5908-6-33 (PMC3080823; doi:10.1186/1748-5908-6-33)
Supplement: Additional File 2 — Excluded studies. Excluded studies with reasons for exclusion [file 1748-5908-6-33-S2.DOC]

**Additional File 2. Excluded Studies**

| **Study ID** | **Reason for exclusion** |
| --- | --- |
| Christianson 1997 | Descriptive |
| **Clemmer 1999** | No control |
| **Conly 1989** | Not changing culture |
| Huq 2000 | Quasi-qualitative case study |
| **Jain 2006** | No control |
| **Larson 1997** | Not changing culture |
| **Leclair 1987** | Not changing culture |
| **Lesmond 2008** | Self-report outcome measures only. Not measuring organisational culture. |
| **Lewis 2008** | Self-report outcome measures only. Not measuring organisational culture. |
| **Lindberg 2005** | No sufficient data points to be re-analysed as an ITS |
| **Lokk 2000** | Not changing culture |
| **Mauno 2006** | Not changing culture |
| **Morris 2007** | Not changing culture |
| **Zazzali 2008** | Qualitative design |
